# The effect of short-term exposure to rural interprofessional work on medical students

**DOI:** 10.5116/ijme.5eb6.81cb

**Published:** 2020-06-24

**Authors:** Ryuichi Kawamoto, Daisuke Ninomiya, Taichi Akase, Asuka Kikuchi, Teru Kumagi

**Affiliations:** 1 Department of Community Medicine, Ehime University Graduate School of Medicine, Ehime, Japan

**Keywords:** Short-term exposure, rural interprofessional, medical students

## To the Editor

In Japan, the shortage and uneven distribution of physicians are well documented. The number of physicians has shown absolute and relative deficiencies,^1^ and Japan is always classified in the group with the lowest number of doctors per patient by the Organization for Economic Co-operation and Development.^2^ The uneven distribution of physicians between rural and urban areas has steadily increased since 1980.^1^^,^^3^

Recently, the importance of rural interprofessional work (RIPW) in rural areas has attracted considerable attention. RIPW affords medical students the opportunity to acquire a broader view of their role as a healthcare professional in a rural setting.^2^^,^^4^^,^^5^ The students are educated in the field by medical and nursing preceptors who work closely with the students. The learning objectives include understanding the principles of collaboration, teamwork, and various roles in the healthcare team within a primary healthcare framework.^6^ Exposure to rural healthcare through an education and training continuum is an important factor in choosing to practice in a rural area.^5^^,^^7^^,^^8^ Additionally, in Japan, exposure to rural healthcare during undergraduate medical education has been demonstrated to be an encouraging factor for the choice to practice rural healthcare in the future.^9^^,^^10^^,^^11^

The Ehime Graduate University School of Medicine Community Medical Laboratory Satellite Centers are located in Nomura-cho (Seiyo City) and Kumakogen Town, which are approximately 60 minutes away from Matsuyama City by road, in the mountainous southwest district of Ehime Prefecture. The populations of these towns (approximately 13,000 and 10,600, respectively) are largely engaged in agriculture and are aging rapidly (aging rate, 37% and 41%, respectively). Medical facilities and specialist healthcare in neighboring areas are scarce. As such, the rural hospitals assume numerous duties. Training sites include hospitals, home-visit nursing offices, elderly care facilities, elderly welfare facilities, daycare centers, day service centers, health and welfare centers, and care facilities for the disabled. It is possible for students in residence to visit all these facilities on a weekly schedule. For all activities, each student is assigned a daily role as a team member and learns while contributing to rural medicine. This short-term exposure enhances the interest and knowledge of students with regard to rural healthcare practices.

The positive response rate for many questionnaires was significantly increased after RIPW. The positive response rates to the statement “I'd like to work at a clinic or a regional core hospital in my future career as my life’s work” and “I know what kind of knowledge is necessary for rural healthcare” were markedly increased after RIPW. However, the rates of agreement to the statements “I think that rural healthcare seems serious” and “when engaged in rural healthcare, I think I will fall behind in medical progress” were significantly decreased after RIPW. The rate of positive responses (“strongly agree”, and “agree”) to the statement “I’d like to participate in rural healthcare in the prefecture in the future” was significantly increased after RIPW, from 69.3% to 80.1%. Medical students with “admission from hometown”, “scholarship for regional duty”, "admission by school recommendation", and "first choice of general medicine/general internal medicine/family medicine" showed significantly higher positive responses for participation in rural healthcare in the prefecture after RIPW. An analysis was performed to determine the motivation of students regarding their participation in rural healthcare in the prefecture after RIPW (adjusted for confounding factors as explanatory variables). The results showed that the items “admission from hometown”, “doctor among parents”, “I think that practicing in rural healthcare is worthwhile”, “I want to work in a rural area in the future”, and “I like to talk with patients” were independently and significantly associated with increased student motivation to participate in rural healthcare in the prefecture. However, the item “experience of admission to another university” was independently associated with decreased student motivation.

Short-term exposure to RIPW affected the knowledge and attitude of medical students towards rural healthcare, and increased their intent to practice in a rural setting. It may be imperative that medical schools prompt medical students to participate in RIPW during clinical training in order to solve the shortage of physicians in rural areas. Future research using longitudinal data will enable us to monitor the relationship between RIPW and subsequent choice to practice rural healthcare.

### Conflict of Interest

The authors declare that they have no conflict of interest.
